# Sex differences in social cognition: Insights from a sample enriched for social dysfunction

**DOI:** 10.1371/journal.pone.0354796

**Published:** 2026-09-10

**Authors:** Kathryn C. Kemp, Scott D. Blain, Saba Zehra, Serena DeStefani, Laura Locarno, Kelly Mathis, Carly A. Lasagna, Costanza Colombi, Cynthia Z. Burton, Aubrey M. Moe, Ivy F. Tso

**Affiliations:** 1 Department of Psychiatry and Behavioral Health, The Ohio State University Wexner Medical Center, Columbus, Ohio, United States of America; 2 Department of Psychiatry, University of Michigan, Ann Arbor, Michigan, United States of America; 3 IRCCS Fondazione Stella Maris, Calambrone, Italy; University of Missouri Columbia, UNITED STATES OF AMERICA

## Abstract

Research suggests there are sex differences in social cognitive abilities, with females typically performing better on tasks involving emotion recognition and mental state attribution. However, many prior studies have demonstrated limited replicability, perhaps due to differences in methods employed or samples studied. Given that social cognition is a key determinant of social functioning, and that social dysfunction represents a transdiagnostic issue in neuropsychiatric populations, understanding sex differences in social cognition among individuals with varying levels of social functioning should broaden our understanding of the etiology, expression, and treatment of psychopathology. The present study examined sex differences in gaze perception, perceptual theory of mind, and social inference abilities in a sample (*n* = 212; 58.5% female) of adolescents and young adults (ages 14–30) ranging from minimal to clinical levels of social dysfunction. Females performed better on tasks involving perceptual theory of mind, social inferencing, and eye gaze perception, and most sex differences remained even after accounting for general cognition, age, and psychopathology. The one exception is that females and males did not differ on a complex, dynamic social inferencing task once accounting for psychopathology. Our findings suggest that females may generally be more sensitive to social signals related to the eyes and may more accurately identify social signals when there is subtle or limited information available. Findings indicate the importance of recognizing sex differences in the presentation of social functioning and, by extension, socially relevant psychopathology. Such differences may have implications for tailoring treatments for social dysfunction.

## Introduction

### General sex differences in cognition and social cognition

Extensive research has investigated the potential role of biological sex in cognitive abilities. Although studies indicate minimal, if any, sex differences in general cognitive ability, some disparities have been observed for more specific cognitive domains (see review by Kheloui et al [1]). For instance, males have consistently demonstrated better performance on spatial tasks, particularly those involving mental rotation [2], whereas females tend to perform better on tests of verbal abilities [3]. Females have also shown superior performance on certain memory tasks [3], but results have been inconsistent and may depend on how the task is presented to participants [4].

Social cognition has emerged as another area of interest in the study of sex differences given its overlapping, yet distinct, features with general cognition (e.g., memory, attention, executive functioning). Coupled with findings that males exhibit more direct aggressive behavior toward others [5] and that females self-report higher levels of empathy [6], prominent theories emphasizing the role of biological sex in social behavior [7] suggest females might perform better in cognitive domains specifically relevant to social interaction and prosocial behavior. Indeed, research has suggested some sex differences when it comes to tests of social cognitive abilities, such as emotion recognition and theory of mind. For example, studies have reported that females generally perform better on tasks involving social processing [8], inferring others’ mental states [9], and identifying facial emotional expressions [10–13]. Moreover, studies examining response time have found that women are both faster and more accurate in discriminating between emotions [12]. This advantage may be partly attributed to women's greater attention to the eye region during face perception [10] and to their lower thresholds for detecting emotions at varying intensities [14].

Although many studies suggest better social cognition in females, other research presents a more complex picture. Vaskinn and colleagues [15] reported limited evidence for sex differences in theory of mind, but stronger evidence for female superiority in emotion recognition across all examined groups. Additionally, although women often demonstrate better performance in emotion recognition tasks, this advantage is not uniform across all studies and conditions [13]. Results vary and are influenced by factors such as age [11] or the specific emotion being assessed [6,16]. For example, Di Tella and colleagues [6] found that females exhibited better recognition of angry faces specifically. Notably, when emotions are presented dynamically (e.g., changes in emotional expression) rather than as static images, sex differences in emotion recognition accuracy sometimes disappear [17]. Thus, task-presentation methods may also play a role in observed sex differences. Additional research using a variety of methods assessing different domains is necessary to clarify sex differences in social cognition.

### Sex differences in social cognition in clinical populations

Although general-population studies of sex differences in social cognition have provided foundational knowledge, understanding sex differences is even more pertinent to clinical populations. Difficulties with social cognition have been consistently observed in a variety of diagnoses, such as schizophrenia and autism spectrum disorder [18], as well as social anxiety disorder (see review by Alvi et al. [19]). Given the prevalence of social cognitive deficits in psychopathology, population-level sex differences in social cognition may be further exaggerated in clinical groups. Nonetheless, examination of clinical data has yielded similarly contradictory evidence regarding sex differences in social cognition.

Pinkham and colleagues [20] assessed social cognition in individuals with schizophrenia across multiple domains, including emotion recognition, theory of mind, and attributional style. Despite employing multiple tests for each domain, they found no significant sex differences. Research on first-episode psychosis has produced mixed results, with Labad et al. [21] finding better performance among females on an emotional intelligence test, whereas other groups have found no sex differences across a variety of other task [22]. Studies on sex differences in social cognition among individuals with autism spectrum disorders have also yielded inconsistent findings. Mattern et al. [23] suggested that girls with autism may exhibit better social cognition and understanding of social causality than boys. Other studies have at times found no sex differences in accuracy on social cognition tasks among people with autism [24,25], although there is evidence of faster reaction times and different patterns of brain activation in females with autism [25]. Lastly, social anxiety is another form of psychopathology that may play a role in sex differences in social cognition. For instance, research has shown that women tend to have higher levels of social anxiety than men [26], leading some to suggest that any observed sex differences in social cognition in general and clinical samples could be more directly explained by differences in social anxiety [27]. Indeed, Di Tella and colleagues [6] found no sex differences in theory of mind when males and females sampled from the general population were matched for social anxiety levels. Because social anxiety is common among clinical populations, it may be an uncontrolled factor that has contributed to conflicting findings in the literature. Furthermore, general cognitive abilities, which overlap to some degree with social cognition [28] and can also be impacted by varying levels of psychopathology, have often gone unaccounted in social cognition research and may play a role in inconsistent findings. Thus, accounting for neurocognition and psychopathology factors may elucidate the role of sex in social abilities specifically.

In sum, although research has revealed differences in social cognition between the sexes, only a limited number of these findings have been consistently replicated, and results may depend upon additional factors such as the specific tasks utilized, general cognitive abilities, and presence of psychopathology in study participants. This underscores the need for further research to clarify the nature and extent of sex differences in social cognition. Furthermore, the transdiagnostic nature of social impairment necessitates examination of sex differences across diagnostic boundaries; such an examination may provide clarification of differences that are not simply attributable to specific diagnostic features.

### Goals and hypotheses

The primary goal of the present study was to clarify sex differences in social cognition using a sample selected for varying levels of social dysfunction (i.e., mild to severe challenges with interpersonal conflict/avoidance, and in developing and maintaining meaningful interpersonal relationships). As noted, lack of clarity regarding sex differences in social cognition may be due to differences in tasks and types of samples. We aimed to address inconsistencies in findings regarding sex differences in social cognition by comparing males and females from a young, transdiagnostic sample in their performance on several social cognitive tasks that vary in complexity. This sample is ideal for examining potential differences, as prominent social difficulties emerge or worsen in adolescence and young adulthood, providing an appropriate age range to observe sex-specific effects. Furthermore, selection based on social dysfunction provided a sample with a range of social cognition difficulties (which are known contributors of social dysfunction). Lastly, examining social cognition transdiagnostically provided a dimensional, nuanced examination of sex differences that are not merely driven by differences in discrete forms of psychopathology.

Specifically, we examined sex differences in gaze perception, mental state attribution, and social inference abilities. These domains were selected because they capture a range of low-level (perceptual) to higher-level (mentalizing, social inference) social cognition. Although there are inconsistencies in the literature, the general trend suggests female superiority on social cognition [e.g., 8,13]. Therefore, we hypothesized that females from a sample spanning the social dysfunction spectrum would demonstrate superior performance on all tests of social cognition. Furthermore, we hypothesized these sex differences would be robust against (or even stronger after accounting for) potentially confounding variables (i.e., visuospatial ability, age, IQ). Lastly, given the transdiagnostic nature of social dysfunction, we examined in an exploratory fashion whether any sex differences in social cognition were impacted by psychopathology factors.

## Methods

### Participants

Participants (*n* = 212; 58.5% female) were recruited via Internet and community advertisements and local clinics to complete an ongoing National Institute of Health (NIH) funded study [29] on mechanisms of social cognition and social dysfunction. Power analysis was conducted in relation to the primary aims of this parent study, which provided a conservatively large sample size for the distinct goals of the present study. Participants self-reported their biological sex. All participants met the following eligibility criteria defined for the broader study: 1) between the ages of 14–30; 2) ability and willingness to provide informed consent; 3) vision equal to or better than 20/30 on the Snellen visual acuity test, with correction if necessary; 4) no significant neurological abnormalities, such as seizure disorder, mass lesions, etc.; 5) no known Mendelian disorder; 6) no active substance use in the past 30 days; 7) IQ ≥ 80. To sample the full range of social functioning, trained interviewers identified participants based on social dysfunction scores as determined by the Mental Illness Research, Education, and Clinical Center’s Global Assessment of Functioning (MIRECC-GAF [30]) and Global Functioning Scale Social subscale (GFS-Social [25]). The MIRECC-GAF and GFS-Social define social dysfunction based on the degree of difficulty with maintenance of meaningful relationships, development of new relationships, and interpersonal conflict or avoidance in the past month. For example, these measures assess the number of close relationships, frequency of social interactions, and frequency of conflicts, social avoidance, and social withdrawal. Both measures were included to increase reliable identification of participants with social dysfunction. Cut-offs defined within the scales were used to select participants. Specifically, participants with a MIRECC-GAF score of 69 (“borderline dysfunctional”) or less or GFS-Social score of 6 (“moderate impairment”) or less were classified as having social dysfunction, whereas scores higher than both these thresholds identified participants with no social dysfunction. Participants identified as having no social dysfunction were eligible for the study if they additionally had no history of mental illness diagnoses based on the Diagnostic and Statistical Manual of Mental Disorders – 5 and were not currently taking any psychotropic medications.

Although participants were not selected based on clinical or diagnostic status, this enriched sample consisted of participants with social anxiety disorder (n = 40), autism spectrum disorder (n = 24), a psychotic spectrum disorder (n = 23), various comorbidities of these diagnoses (n = 53), subclinical levels of these diagnoses (n = 25), as well as a comparison group (n = 47) identified as having no social dysfunction. Examining across these diagnostic groups that are defined by the shared feature of social dysfunction (as opposed to splitting by group) offered a meaningful and statistically robust method to determining sex differences in social cognition. See S1 and S2 Tables for demographic information and descriptive statistics for the total sample and summarized separately for males and females.

### Measures and procedures

Participants completed all tasks as part of a larger eye-tracking and neuroimaging study (see Tso et al. [29] for approved NIH proposal) completed at the University of Michigan (U of M) and The Ohio State University (OSU). The project may be accessed on the National Institute of Mental Health’s Data Archive at https://nda.nih.gov/edit_collection.html?id=3489. The present study specifically examines performance on a variety of behavioral tasks included in the parent study that capture low-level (perceptual) and higher-level (mentalizing, social inferencing) social cognition. Inclusion of tasks capturing these different levels of complexity allowed us to discern at which levels sex differences emerge. Note that the larger study from which data for the current analyses were drawn collected information on sex assigned at birth but did not assess gender identity. The study was approved by the University of Michigan’s Institutional Review Board and The Ohio State University’s Institutional Review Board. The data collection period for this study began at U of M on 17/05/2021 and at OSU on 18/06/2024. Data collection for both sites ended on 16/12/2025 for the present study. Participants provided written informed consent, and for minors who assented to study participation, their parent/guardian provided written informed consent. All participants completed psychopathology assessments and behavioral tasks (S3 Text). A subset of participants completed additional behavioral tasks administered in the context of neuroimaging. All participants received compensation for their time.

A variety of questionnaire and clinician-rated measures (S4 Text) were administered to capture three relevant psychopathology dimensions (psychosis proneness, autism, social anxiety) for the purposes of analyses examining sex differences over-and-above these characteristics. To examine psychosis proneness, participants completed the Peters Delusion Inventory (PDI) [31], Cardiff Anomalous Perception Scale (CAPS) [32], and Scale for the Assessment of Negative Symptoms (SANS) [33]. For consistency with other psychopathology measures, the total number of endorsed items on the PDI and CAPS, and not other subscores (e.g., distress level), were used for analyses. To examine autism traits, participants completed the Autism Spectrum Quotient (AQ) [34] and the Autism Diagnostic Observation Schedule Second Edition (ADOS) [35]. The revised algorithm for the ADOS [35] specifically was used for analyses. To examine social anxiety, participants completed the Social Phobia Inventory (SPIN) [36] and Social Anxiety Disorder Dimensional Scale (SADD) [37], and item totals on these measures were computed. Multiple measures of each trait dimension were included to increase reliability and comprehensiveness of content coverage.

**Fig 1:**
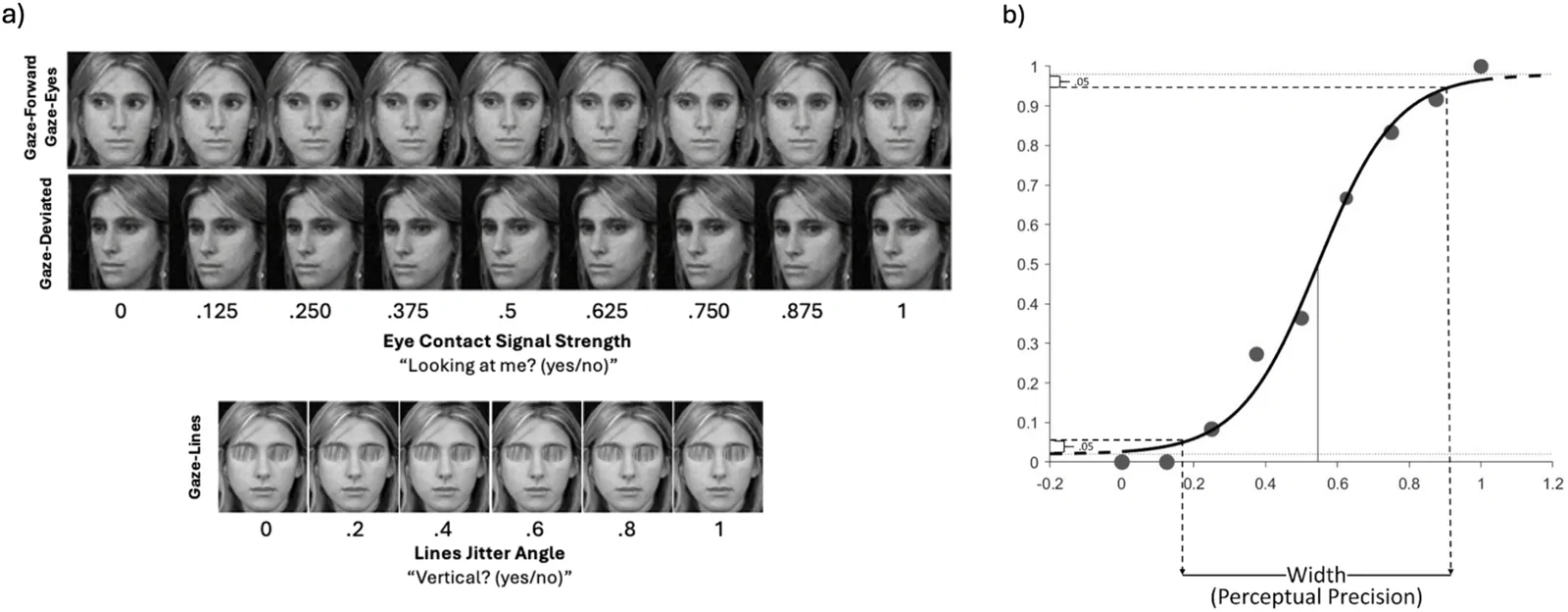
Gaze perception task. *Note*. Adapted from Fig 1 in Lasagna et al. [38] under a CC BY 4.0 license. Participants are shown a series of face images [40] with varying signal intensities. The nature of the signal intensity depends on the particular task condition (a). For the Gaze-Forward, Gaze-Eyes, and Gaze-Deviated conditions, they are asked to determine if each face is looking at them (yes/no). In these conditions, the eye contact signal intensity ranges from weak (i.e., clearly not looking at me) to strong (i.e., clearly looking at me) in gradual increments. Gaze-Forward (non-scanner) and Gaze-Eyes (scanner) both had forward-facing head orientations, whereas Gaze-Deviated (non-scanner) had deviated-facing head orientations. For the Gaze-Lines condition, they are asked whether each set of lines superimposed over the eye region is vertical (yes/no). In these conditions, the “vertical signal intensity” ranges from weak (i.e., lines clearly slanted) to strong (i.e., clearly vertical) in gradual increments. To index participant’s perceptual precision for each condition, their endorsement rates were modeled as a logistic function of signal intensity and a “width” metric was computed as the difference between signal intensities at the points where the participant responded “yes” 5% versus 95% of the time. A similar psychophysical precision metric (i.e., the width of the logistic function between 5% and 95% ‘yes’ responses) was computed for both the gaze and lines conditions, allowing us to control for non-social perceptual processes. (b). Smaller width values reflect greater precision, showing that the participant is more sensitive to small changes in gaze or line angles.

To examine low-level social cognitive abilities, participants completed several versions of the Gaze Perception Task [38] (Fig 1). This task provided a measure of one’s ability to discern the direction of others’ gaze, which is a fundamental skill that supports higher-level social abilities [39]. First, participants completed a behavioral protocol, which included two conditions (Gaze-Forward and Gaze-Deviated) that capture perceptual social cognition. For both conditions, participants were shown a series of face images and asked to determine if each face was looking at them (yes/no). For Gaze-Forward, face images were directed toward the center of the screen. For Gaze-Deviated, face images were directed away from the center of the screen. There were nine different signal intensities for the face stimuli, with eye-contact strength that varied from fully direct to fully averted in small increments (Fig 1a). Participants completed a total of 108 Gaze-Forward trials in one block and 108 Gaze-Deviated trials in a second block.

A subset of participants went on to complete a neuroimaging protocol, which included two conditions of the Gaze Perception Task (Gaze-Eyes and Gaze-Lines) and provided additional information about perceptual social cognition. Note again that only behavioral data were examined in this study. Although administered during neuroimaging, Gaze-Eyes was the same task as Gaze-Forward described above. For Gaze-Lines, participants viewed a series of face images with sets of lines imposed over the face’s eye region, and responded whether these lines were vertical (yes/no). There were six different signal intensities with lines varying from fully vertical to diagonally tilted (Fig 1a). The inclusion of Gaze-Eyes and Gaze-Lines conditions provided the opportunity to examine gaze perception while controlling for visuospatial ability. The Gaze-Lines condition serves as a control condition for Gaze-Eyes, assessing basic visuospatial abilities with similar difficulty to Gaze-Eyes but without access to gaze information. Participants completed a total of 108 Gaze-Eyes trials and 72 Gaze-Lines trials, spread over three runs, each with four gaze and four lines blocks.

Using principles of psychophysics, the Gaze Perception Task was analyzed to provide an estimate of perceptual precision on each condition. First, endorsement rates (i.e., proportion of “yes” responses) were computed for each signal intensity (i.e., gaze angle or line angle). Then, endorsement rates were modeled as a logistic function of signal intensity, using Bayesian estimation implemented through the psignifit 4 toolbox [41] for MATLAB. To index precision, we then computed the widths of the psychometric perception curve of each participant across all conditions, defined as the difference between signal intensities at the points where the participant responded “yes” 5% versus 95% of the time (Fig 1b). Smaller width values reflect greater precision, showing that the participant is more sensitive to small changes in gaze or line angles. More complete details of this fitting procedure are detailed in S5 Text. A similar psychophysical precision metric (i.e., the width of the logistic function between 5% and 95% ‘yes’ responses) was computed for both the gaze and lines conditions, allowing us to control for non-social perceptual processes. Perceptual precision on this task was used as a metric of social cognitive ability in this study because it has been shown to correlate highly with accuracy on other social cognition measures [38].

Lastly, participants completed two behavioral tasks capturing different aspects of mental state attribution abilities. These tasks captured more advanced social cognition skills beyond those of the Gaze Task and thus allowed examination of different levels of complexity at which sex differences may occur. Participants completed the Reading the Mind in the Eyes test (RME) [42], which examines perceptual theory of mind. Specifically, the RME builds upon our examination of low-level perceptual abilities by requiring interpretation of social information conveyed by a pair of eyes. This task consists of 36 pictures of eyes portraying different emotional or mental states, and participants select the appropriate state being exhibited from four multiple-choice options. Accuracy was computed as total correct answers. Participants additionally completed The Awareness of Social Inference Test (TASIT) [43], which provides an additionally complex evaluation of mental state attribution as it requires the simultaneous integration of social information exhibited across modalities (visual/movements, speech/tonal) to make social inferences. The TASIT is comprised of 18 videos that range from 15 to 60 seconds in length. After each video, participants answered four yes/no questions about the characters’ actions and mental states. Accuracy was computed as a percentage for overall performance.

### Statistical analyses

A series of regression analyses were used to examine the effect of sex (female = 0, male = 1) on performance for each behavioral task. A visuospatial control (Gaze-Lines) was included as a covariate in the model predicting Gaze-Eyes, but not Gaze-Forward and Gaze-Deviated (as only a subset of participants completed neuroimaging). Thus, the Gaze-Forward and Gaze-Deviated conditions provided an initial assessment of sex differences in perceptual social cognition, whereas the neuroimaging tasks (Gaze-Eyes and Gaze-Lines) allowed us to account for visuospatial abilities and provide an estimate specific to social cognitive performance.

To examine whether sex-difference results were consistent after accounting for other relevant variables, we reran the same regression analyses with two different sets of covariates in the model. First, we accounted for IQ and age to ensure that the observed sex differences were not solely due to differences in intellectual functioning or age (given the different developmental periods included in the age range of this sample). Second, in addition to IQ and age, we included psychopathology indicators as covariates. Because there are both known sex differences and social cognitive deficits in psychopathology, accounting for these indicators served as additional sensitivity analyses to examine whether any observed sex differences were driven by psychopathology. To account for psychopathology, we conducted a principal components analysis (PCA) based on our seven measures of dimensional psychopathology (AQ, ADOS, SPIN, SADD, SANS, PDI, and CAPS) and extracted factors to be included as additional covariates in our regression models. Participants were included in the PCA (*n* = 185) if they completed at least 80% of the psychopathology measures in order to increase reliability of imputation. Missing values among these participants were imputed using Bayesian PCA (pcaMethods package in R).

## Results

### Sex differences in social cognition tasks

**Fig 2:**
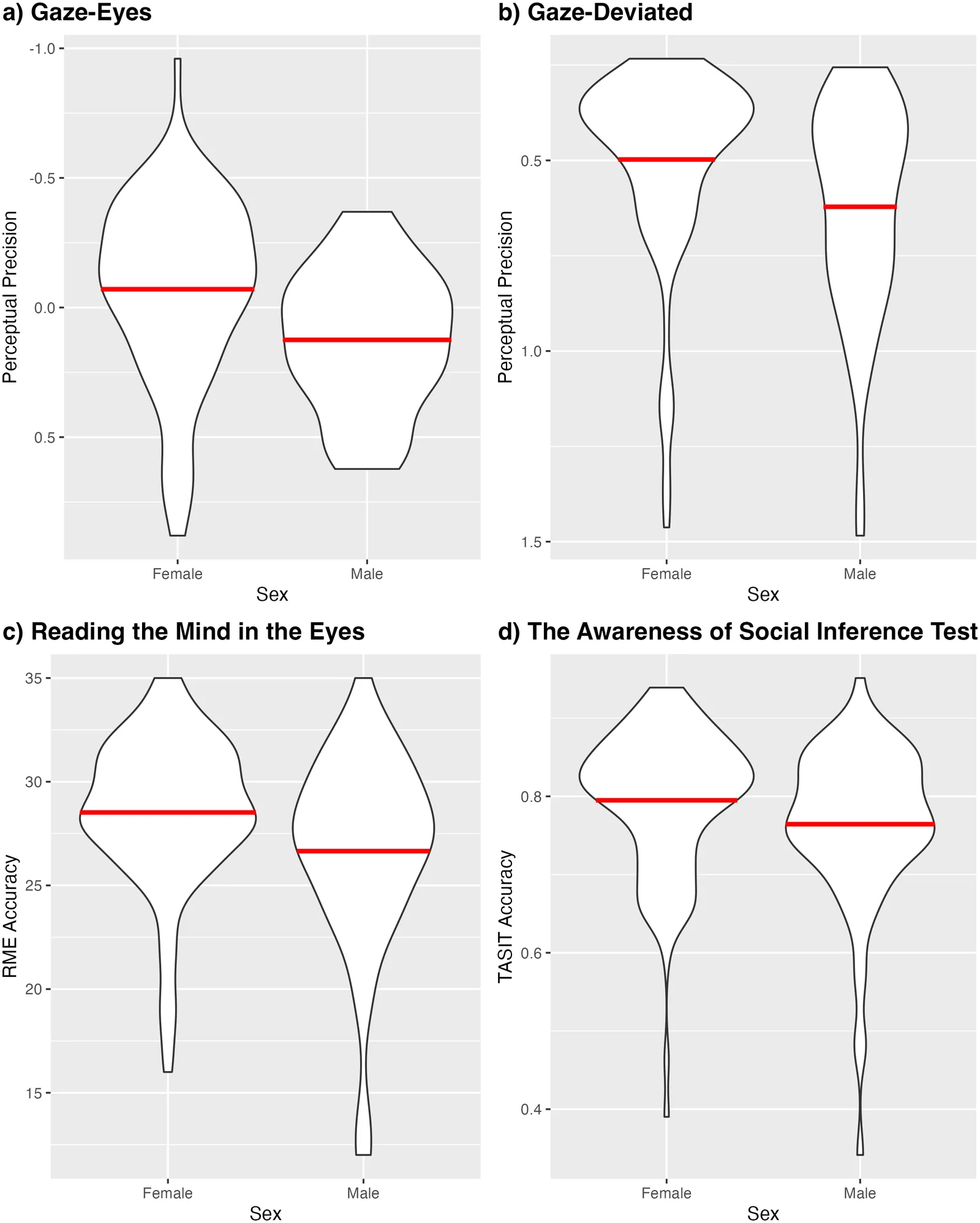
Violin plots showing performance on social cognition tasks by sex. Note: 0 = female, 1 = male; red bars represent group mean on each task. a) Y-axis represents participants’ psychophysical width for the task, controlling for the Gaze-Lines condition (residualized variable), y-axis is flipped to represent how lower values indicate greater precision; b) Y-axis represents participants’ psychophysical width for the task, without controlling for other variables (non-residualized variable), y-axis is flipped to represent how lower values indicate greater precision.

All results from the regression analyses predicting social cognition by sex are displayed in Table 1. Violin plots provide a visual representation of observed sex differences (Fig 2). There were significant sex differences on the RME (*β* = −.22; *p* = .002), TASIT (*β* = −.15; *p* = .039), and Gaze-Deviated tasks (*β* = .23; *p* = .002), such that females exhibited better performance than males. Additionally, females showed greater perceptual precision on Gaze-Eyes both when Gaze-Lines was included as a covariate (*β* = .25; *p* = .004) and when Gaze-Lines was removed as a covariate (*β* = .25; *p* = .010; S6 Table). All results were retained, and several results were stronger, when age and IQ were included as covariates. There were no significant sex differences on Gaze-Lines (*β* = −.01; *p* = .906) or Gaze-Forward (*β* = .08; *p* = .287).

**Table 1:** Effects of sex on social cognition.

Criterion Variable		No Demographic Covariates				Covarying Age, IQ			
	*n*	*b*	*β*	*t*	*p*	*b*	*β*	*t*	*p*
Gaze Perception Task – No Scanner									
Gaze-Forward	184	.07	.08	1.07	.287	.06	.07	1.03	.307
**Gaze-Deviated**	**184**	**.12**	**.23**	**3.13**	.002**	**.12**	**.22**	**3.11**	.002**
Gaze Perception Task – Scanner									
**Gaze-Eyes**^**a**^	**108**	**.20**	**.25**	**2.99**	.004**	**.20**	**.26**	**3.18**	.002**
Gaze-Lines	108	−.01	−.01	−.12	.906	−.01	−.01	−.13	.895
**Reading the Mind in the Eyes**	**195**	**−1.86**	**−.22**	**−3.09**	.002**	**−1.88**	**−.22**	**−3.13**	.002**
**The Awareness of Social Inference Test**	**181**	**−.03**	**−.15**	**−2.08**	.039*	**−.03**	**−.15**	**−2.09**	.038*

* p < .05

** p < .01.

*Note.* Sex was used as independent variable (females = 0, males = 1). ^a^When predicting performance on Gaze-Eyes, we controlled for performance on Gaze-Lines (but not vice versa). **Lines in bold represent a significant sex difference (favoring females).**

### Sex differences over-and-above psychopathology

PCA provided a useful method of parsimoniously integrating distinct but overlapping psychopathology constructs. Examination of scree plots from parallel analysis indicated there are three components in the seven measures of dimensional psychopathology. These three components accounted for 83% of the total variance in a PCA (S7 Table). The first component had the strongest loadings from the SPIN, SADD, and AQ. Conceptually, the self-report of internal experiences regarding social situations appears to be the shared feature characterizing this component. Namely, the SPIN and SADD measure self-reported affective (i.e., anxious) reactions to social situations. The AQ measures a variety of autism features, but focuses on internal experiences such as social preferences and reactions shared with social anxiety. Thus, we conceptualized this component as representing self-reported affective experiences in social situations and now label it as “Social Affect.” The second component had the strongest loadings from the CAPS and PDI. Given these measures’ examination of positive symptoms characteristic of psychosis-spectrum disorders, we now label this component as “Psychosis Proneness.” The third component was labeled as “Low Social Reciprocity” given its strongest loadings came from the SANS and ADOS.

Separation of the two autism measures (AQ and ADOS) between Social Affect and Low Social Reciprocity may reflect measurement similarities with other indicator variables (i.e., questionnaire versus clinician ratings). Furthermore, the composite variable for the ADOS, which was calculated using the recommended revised algorithm [35], emphasizes social communicative skills over other features of autism. These experiences can be phenotypically similar to negative symptoms of psychosis, which include indicators of diminished social communication. Thus, this data-driven approach of reducing psychopathology dimensions appears appropriate for parsimoniously accounting for the shared and distinct features across socially relevant psychopathology (e.g., in contrast to accounting for group status, which may obscure experiences that cut across disorders). Notably, there were significant sex differences in the psychopathology factors (S8 Table), with females scoring higher on Social Affect and males more strongly exhibiting Low Social Reciprocity. There were no sex differences in Psychosis Proneness in this sample.

Results from adding these psychopathology components as covariates (S9 Table) in the regression analyses were mostly consistent with results when only age and IQ were included in the model. That is, females still exhibited better performance on RME (*β* = −.19; *p* = .019), Gaze-Eyes (*β* = .32; *p*=<.001), and Gaze-Deviated (*β* = .20; *p* = .014) tasks. Thus, sex differences exhibited in these tasks do not appear to be solely attributable to assessed forms of psychopathology. However, sex no longer predicted TASIT performance (*β* = −.02; *p* = .278), suggesting that psychopathology may play a role in sex differences in higher-level social cognitive tasks.

## Discussion

The goal of the present study was to examine whether there are sex differences in social cognition in individuals with varying levels of social functioning. Although females generally outperformed men on social cognition tasks of varying complexity, females more consistently exhibited better performance on tasks in which social information is communicated via the eyes. Specifically, females more accurately identified internal states associated with eyes, and results favored females in the ability to precisely discern whether a pair of eyes is looking at them. These results appear consistent with findings that females attend more to the eye region when processing faces [10], which may enhance social processing among females. Most significant sex differences held when controlling for potentially confounding variables such as intellectual ability, age, and psychopathology.

First, females exhibited strengths in a relatively basic, perceptual-based social cognitive task—eye-gaze processing. Females completed the Gaze Perception Task’s forward-facing eyes condition both during neuroimaging (Gaze-Eyes; for which a visuospatial control was also completed) as well as outside of the scanner (Gaze-Forward; for which no control was completed). Interestingly, the scanner-based Gaze-Eyes task revealed a significant sex effect favoring females regardless of whether visuospatial ability was controlled, whereas the behavioral Gaze-Forward task showed no significant sex effect despite a larger sample. This pattern may reflect contextual factors: the controlled scanner environment likely reduces extraneous variance (e.g., movement, distraction), potentially enhancing detection of subtle perceptual differences. Additionally, the inclusion of a visuospatial control task in the scanner session may have oriented participants toward fine-grained visual discrimination. Importantly, controlling for visuospatial ability did not impact the sex effect in the Gaze-Eyes condition, suggesting that females’ superior performance on eye-gaze processing is not simply attributable to differences in basic visual processing. These findings highlight both the robustness of sex-related differences in gaze perception precision and the importance of measurement context in detecting small effects. Furthermore, females exhibited greater perceptual precision than males on a more challenging condition of the Gaze Perception Task, in which the presented faces were deviated from the center of the screen. Thus, females were shown to exhibit strengths in a basic social-perception skill that supports more complex social cognition and communication [44].

One such form of complex social cognition that may be supported by gaze-perception precision is mental state attribution, and results from the present study converged for two tasks varying in complexity. Specifically, we observed significant female superiority in performance on both the RME and TASIT. Whereas the RME uses static stimuli (images of the eye region of faces) to probe identification of a mental state or emotion, the TASIT uses dynamic stimuli (videos) and requires integrating social information exhibited across modalities (visual/movements, speech/tonal) over time to make social inferences. Thus, females appear better able to discern mental states both from a pair of eyes when isolated from other social information, and possibly also when integrating a variety of social information simultaneously.

Our significant findings for the TASIT contrast with prior findings that the TASIT is not associated with sex differences [45] as well as research suggesting there are no sex differences on accuracy for social cognitive tasks that use dynamic stimuli [e.g., 17]. However, the sex effect in the current study was no longer significant once accounting for psychopathology that is defined by social difficulties, suggesting that psychopathology may better explain sex differences in social inferencing. The challenge of completing a high-level task may thereby be better determined by the specific features of psychopathology rather than sex. Notably, the different social cognition tasks examined in this study vary in degrees of ecological validity, with the TASIT being the most representative of everyday social interactions. Thus, males and females generally may perform comparably when a variety of social information is present (as in most real-world social interactions), which could reflect the ability to call upon multiple sources of information to compensate for deficits in specific social areas and thereby increase accuracy. Yet, accuracy for males may diminish when less information is available, as observed on tasks that isolate processing of eyes presented in static images. The combination of results that females outperform males on both eye-gaze perceptual precision and a mental state attribution task involving the eyes could indicate that females are overall more sensitive to subtle social cues, especially on tasks that attend to the eyes. Future research may benefit from isolating other types of social signals (e.g., body movement, speech) to determine whether other specific mechanisms contribute to sex differences in social cognition.

Furthermore, inconsistent evidence of sex differences in social cognition in prior research may be related to differences in the focus or complexity of the social stimuli used across studies. Complex tasks that recruit multiple cognitive processes (e.g., memory, attention, basic visual perception) offer better ecological validity, as performing well socially in real life likely also involves these other cognitive abilities. However, complex tasks may also mask the contribution of discrete social cognitive skills, making it difficult to isolate which specific abilities drive performance. For example, the TASIT requires retrospective recall to answer questions following each video stimulus (e.g., “Is she trying to say she’s happy to help out with a salad?”). Thus, the difference in results on the TASIT once accounting for psychopathology may be related to memory biases or deficits that occur in clinical populations that can affect performance, whereas performance on the RME and Gaze Perception Task require less recruitment of other cognitive abilities due to the simultaneous presentation of stimulus and measurement of response. Future studies should directly evaluate whether sex differences in social cognition are moderated by the complexity or type of task stimuli.

### Limitations

Although our results align well with the literature examining sex differences in social cognition, our sample differs from those of previous research. Specifically, our sample contains more females than males (58.5% versus 41.5% of sample) and contains participants selected based on varying levels of social dysfunction (thus representing a broad range of psychopathology). The reliability and interpretation (and therefore generalizability) of our results may be obscured by differences in precision of estimates in males versus females as well as the heterogeneity of psychiatric conditions. However, psychopathology research (particularly psychosis and autism) has disproportionately examined males and has typically restricted study inclusion to non-clinical controls and participants with a specific diagnosis. Although inconsistencies between the literature and the results of the present study may be reflective of differences in sample characteristics, we emphasize that our sample characteristics are an advantage of the current study. Namely, prior studies with clinical samples may have offered less precise estimates of female performance. Furthermore, although social anxiety, psychosis-spectrum disorders, and autism represent distinct conditions, transdiagnostic examinations following recruitment based on social dysfunction levels provides variability in performance and thus could enhance detection of sex differences if present. Examining sex differences in discrete diagnostic categories or in alternate PCA configurations (e.g., using the ADOS and AQ alone to create an autism component) may be important to researchers or clinicians who are working with a specific patient population and wish to understand how social cognition differs by sex in that population (e.g., for treatment modification purposes).

The present study specifically asked participants to report their biological sex yet did not inquire about gender identity. It can be challenging to compare the present study to prior “sex difference” research given inconsistencies in operationalization; that is, prior research examining sex differences has often conflated sex and gender [46]. Future research could attempt to separately measure sex and gender (either categorically or dimensionally), to elucidate any possible dissociable contributions to social cognition. Lastly, it is important to note that different developmental periods (adolescence and young adulthood) do play a role in sex differences in social cognition. For example, pubertal timing has been related to maturation of social brain structures [47]. Although we controlled for the impact of age given the span of our sample across different developmental periods, we are unable to examine the trajectory of sex differences in social cognition across the lifespan. Future research may benefit from examining whether sex influences the development or deterioration of social cognitive abilities at different points in development, including mid and late life (which were not captured in this study). Relatedly, the current sample spans a developmental window during which social cognitive abilities are still maturing, which may influence the magnitude or direction of observed sex differences.

Note that we conceptualized accuracy (or perceptual precision) on tasks as our primary indicator of social cognitive performance in the present study. However, this may be a limitation as there are other metrics indicative of social cognitive ability, such as reaction time and bias. For example, two participants may respond accurately to a stimulus, yet one participant may respond immediately whereas the second participant requires additional time. An accuracy metric would indicate that these two participants are equally adept at the task, but reaction time would suggest more efficient social cognitive processing in the former participant. Computational models integrating relevant performance metrics [48] would offer further insight into social cognitive abilities between sexes.

### Future implications and conclusions

Research has suggested there may be sex differences in social cognition, which play an important role in social functioning. Thus, examining sex differences in social cognition may provide a fruitful area of study to better understand disease processes and facilitate treatment for disorders that are both characterized by social dysfunction and exhibit sex differences in prevalence and expression. For example, given the finding that females are consistently more accurate with limited social information, females may benefit more from coaching that focuses on very specific social information, whereas males may benefit from incorporation of a variety of social and broader cognitive/perceptual information in treatments targeting social difficulties. The present study adopted a transdiagnostic approach to examining sex differences in social cognition by enriching the sample based on social dysfunction levels rather than diagnostic status. Given the connection between social cognition and social functioning, these sampling procedures provide more information for investigating sex differences in social cognition compared to recruiting based on static, categorical, and stratified diagnoses within our current diagnostic system that rely on arbitrarily defined symptom counts. Yet, this investigation may also extend our understanding of specific disorders often characterized by social cognitive and functioning deficits, such as schizophrenia, autism, and social anxiety. All in all, our results provide support that, even when both males and females are selected for varying levels of social difficulties, females exhibit better skills in some domains of social cognition. Nevertheless, future studies may benefit from inclusion of more severe clinical presentations and incorporation of more nuanced computational models of social cognition.

## Supporting information

S1 Table Demographic characteristics. (DOCX)

S2 Table Descriptive statistics by sex. (DOCX)

S3 Text Study protocol. (DOCX)

S4 Text Description of psychopathology measures. (DOCX)

S5 Text Gaze Task psychometric curve fitting. (DOCX)

S6 Table Effects of sex on gaze perception – Model without covarying condition. (DOCX)

S7 Table Factor loadings for the principal components analysis of dimensional psychopathology measures. (DOCX)

S8 Table Psychopathology factors by sex. (DOCX)

S9 Table Effects of sex on social cognition over-and-above psychopathology. (DOCX)
