# Supplementary material for: Sex differences in social cognition: Insights from a sample enriched for social dysfunction
Source: PLoS One. 2026 Sep 10;21(9):e0354796. doi: 10.1371/journal.pone.0354796 (PMC13561320; doi:10.1371/journal.pone.0354796)
Supplement: Supplementary file 1 [file pone.0354796.s001.docx]

|  |  | **Total** | | **Female** | | **Male** | |
| --- | --- | --- | --- | --- | --- | --- | --- |
|  |  | *n* | % | *n* | % | *n* | % |
| Group |  |  |  |  |  |  |  |
|  | Total | 212 | - | 124 | 58.5 | 88 | 41.5 |
|  |  |  |  |  |  |  |  |
|  | Patient |  |  |  |  |  |  |
|  | SAD only | 40 | 18.9 | 33 | 26.6 | 7 | 8.0 |
|  | ASD only | 24 | 11.3 | 8 | 6.5 | 16 | 18.2 |
|  | PSD only | 23 | 10.8 | 10 | 8.1 | 13 | 14.8 |
|  | Comorbid | 53 | 25.0 | 31 | 25.0 | 22 | 25.0 |
|  | Subclinical | 25 | 11.8 | 13 | 10.5 | 12 | 13.6 |
|  | Control | 47 | 22.2 | 29 | 23.4 | 18 | 20.5 |
| Race |  |  |  |  |  |  |  |
|  | Asian | 32 | 15.1 | 19 | 15.3 | 13 | 14.8 |
|  | Black | 14 | 6.6 | 9 | 7.3 | 5 | 5.7 |
|  | White | 150 | 70.8 | 89 | 71.8 | 61 | 69.3 |
|  | Native Hawaiian or Pacific Islander | 1 | 0.5 | 0 | 0 | 1 | 1.1 |
|  | More than one race | 14 | 6.6 | 7 | 5.6 | 7 | 5.7 |
|  | Unknown/Prefer not to answer | 1 | 0.5 | 0 | 0 | 1 | 1.1 |
|  |  |  |  |  |  |  |  |
| Ethnicity | Hispanic | 16 | 7.5 | 6 | 4.8 | 10 | 11.4 |
|  | Non-Hispanic | 196 | 92.5 | 118 | 95.2 | 78 | 88.6 |
|  |  |  |  |  |  |  |  |
| Medication |  |  |  |  |  |  |  |
|  | Anti-Psychotics | 43 | 20.3 | 25 | 20.2 | 18 | 20.5 |
|  | Anti-Cholinergic | 5 | 2.4 | 3 | 2.4 | 2 | 2.3 |
|  | Anti-Depressants | 85 | 40.1 | 56 | 45.2 | 29 | 33.0 |
|  | Benzodiazepines | 19 | 9.0 | 14 | 11.3 | 5 | 5.7 |
|  | Stimulants | 45 | 21.2 | 29 | 23.4 | 16 | 18.2 |
|  | Mood Stabilizers | 27 | 12.7 | 19 | 15.3 | 8 | 9.1 |
|  | Non-Psychotropic | 114 | 53.8 | 77 | 62.1 | 37 | 42.0 |
|  | None | 60 | 28.3 | 27 | 21.8 | 33 | 37.5 |

*Note.* SAD = Social Anxiety Disorder; ASD = Autism Spectrum Disorder; PSD = Psychotic Spectrum Disorder; Percentages are calculated as the proportion of the respective sample reported.
