# Supplementary material for: Sex differences in social cognition: Insights from a sample enriched for social dysfunction
Source: PLoS One. 2026 Sep 10;21(9):e0354796. doi: 10.1371/journal.pone.0354796 (PMC13561320; doi:10.1371/journal.pone.0354796)
Supplement: Supplementary file 2 [file pone.0354796.s002.docx]

|  | **Female** | | **Male** | |  |  |
| --- | --- | --- | --- | --- | --- | --- |
|  | *M* | *SD* | *M* | *SD* | *t* | *p* |
| Age (years) | 23.10 | 4.26 | 22.59 | 4.85 | .79 | .433 |
| FSIQ2 | 113.71 | 12.04 | 113.05 | 15.02 | .34 | .733 |
| SES Scores | 2.51 | .82 | 2.49 | .97 | .16 | .875 |
| Education (years) | 14.96 | 2.87 | 13.58 | 3.05 | **3.36** | **<.001***** |
| Psychopathology Traits |  |  |  |  |  |  |
| AQ Total Score | 26.47 | 10.01 | 25.23 | 8.83 | .89 | .375 |
| ADOS Total Score | 4.20 | 4.46 | 5.77 | 5.29 | **-2.12** | **.035*** |
| SADD Total Score | 14.94 | 10.14 | 12.48 | 9.14 | 1.76 | .080 |
| SPIN Total Score | 32.85 | 17.57 | 26.79 | 15.78 | **2.43** | **.016**** |
| CAPS Total Score | 5.15 | 5.94 | 5.05 | 5.78 | .12 | .905 |
| SANS Total Score | 2.42 | 2.42 | 3.48 | 2.84 | **-2.72** | **.007**** |
| PDI Total Score | 3.33 | 3.63 | 4.49 | 4.48 | -1.90 | .060 |
| Social Functioning |  |  |  |  |  |  |
| WSAS Total Score | 7.48 | 4.87 | 7.49 | 4.75 | -.02 | .985 |
| MIRECC Social | 67.75 | 14.79 | 66.94 | 15.89 | .37 | .709 |
| GFS Social | 6.61 | 1.58 | 6.31 | 1.70 | 1.33 | .185 |

*Note.* Sex was coded as female = 0, male = 1; FSIQ2 = Full Scale Intelligence Quotient (Verbal and Matrix Reasoning from the Wechsler Abbreviated Scale of Intelligence, McCrimmon & Smith, 2013*; possible range: 45-160); SES = Socioeconomic Status (Self-Report based on the following guidelines – 1: Family of wealth, education, top-rank social prestige; 2: Professional or high level managerial position; adults hold college or advanced degrees; 3: Small businessperson, white collar and skilled workers, high school graduates; 4: Semi-skilled workers, laborers, education below secondary level; 5: Unskilled and semi-skilled workers, elementary education); AQ =Autism Spectrum Quotient; ADOS = Autism Diagnostic Observation Schedule; SADD = Social Anxiety Disorder Dimensional Scale; SPIN = Social Phobia Inventory; CAPS = Cardiff Anomalous Perception Scale; SANS = Scale for the Assessment of Negative Symptoms; PDI = Peters Delusion Inventory; WSAS = Work and Social Adjustment Scale; MIRECC = Mental Illness Research Education and Clinical Center; GFS = Global Functioning Scales

* McCrimmon AW, Smith AD. Review of the Wechsler abbreviated scale of intelligence, (WASI-II).
