# Supplementary material for: Sex differences in social cognition: Insights from a sample enriched for social dysfunction
Source: PLoS One. 2026 Sep 10;21(9):e0354796. doi: 10.1371/journal.pone.0354796 (PMC13561320; doi:10.1371/journal.pone.0354796)
Supplement: Supplementary file 3 [file pone.0354796.s003.docx]

| **Protocol Title:** | Disrupted eye gaze perception as a biobehavioral marker of social dysfunction: An RDoC investigation |
| --- | --- |
| **Principal Investigator:** | Ivy Tso, PhD  Associate Professor    The Ohio State University Wexner Medical Center  Department of Psychiatry and Behavioral Health  1960 Kenny Road  Room 208  Columbus, OH 43201    Ivy.Tso@osumc.edu |

**Introduction**

Social dysfunction significantly contributes to disability across mental illnesses, undermining patients’ capacities for employment, independent living, and maintaining meaningful relationships. Currently available medications fail to address social dysfunction. Although psychosocial therapies are helpful, they take substantial time, are hard to access, and are difficult to adapt to different diagnoses. To improve the outcome of mental illnesses, treatments that can efficiently reduce social deficits spanning many diagnostic categories are needed. The first steps to achieving this goal are to identify key markers of social functioning across mental illnesses and to understand their underlying mechanisms.

Eye gaze perception, the ability to discriminate others’ gaze direction, is a potential biomarker of social functioning that spans multiple diagnoses. Primate literature shows that gaze perception is a basic building block supporting higher-level social communication and social development, and its disruptions leads to social dysfunction. Gaze perception is subserved by complex interactions among multiple brain networks, including the visual, salience, and fronto-parietal control networks. Our research shows that gaze perception is disrupted in schizophrenia and is associated with deficits in important social cognitive areas (e.g., emotion discrimination, theory of mind, emotion regulation). Our behavioral and brain connectivity findings indicate that both bottom-up (sensory processing) and top-down (prefrontal modulation of the visual system) deficits are involved. This lends support to our central premise that gaze perception has a direct impact on real world functioning and investigating its neural underpinnings can reveal which neural circuits to target in future interventions. Abnormal gaze perception is not specific to schizophrenia as it has also been observed in other psychosis-spectrum conditions (e.g., bipolar disorder, schizotypal) and non-psychosis disorders (e.g., autism-spectrum disorders, social phobia), all of which exhibit significant social dysfunction. What remains to be determined is when abnormal gaze perception emerges, how it evolves with illness stage, and whether the same mechanisms are involved in other mental illnesses with prominent social deficits. These studies are needed to develop effective treatments for social dysfunction that can be applied to different psychiatric diagnoses. The overall objective of this project is to test the hypothesis that eye gaze perception is a common mechanism of social dysfunction across psychiatric disorders.

**Specific Aims:**

Aim #1: Determine the generality of gaze perception disturbances in psychiatric patients with prominent social dysfunction.

Aim #2: Map behavioral indices of gaze perception disturbances to *dimensions* of psychiatric phenotypes and core functional domains.

Aim #3: Identify the neural correlates of altered gaze perception in psychiatric patients with social dysfunction using fMRI.

**Significance**

This study will identify the specific basic deficits, clinical profile, and underlying neural circuits associated with social dysfunction. These will guide new treatments combining specific cognitive training (visual and/or self-referential processing) and brain stimulation (e.g., TMS, HD-tDCS) to improve social functioning in patients.

**Study Design**

We will recruit 200 patients having various degrees of social dysfunction (including but not limited to psychosis-spectrum disorders, autism-spectrum disorders, and social anxiety) and matched healthy controls. We will recruit participants between the ages of 14-30, where most mental illnesses emerge and escalate. All participants will complete a comprehensive battery to characterize psychiatric phenotypes, cognition, social cognition, and community functioning. Eye gaze perception will be assessed using a psychophysical task, and two metrics indexing independent cognitive processes involved in gaze perception (precision, self-referential bias) will be derived using hierarchical Bayesian modeling. This cross-sectional naturalistic study involves 2 sessions. Participant’s psychiatric phenotypes, cognition, social cognition, and community functioning will be dimensionally characterized. Eye gaze perception will be assessed using a psychophysical task, and two metrics (precision, self-referential bias) that respectively tap into gaze perception disturbances at the visual perceptual and interpretation levels, independent of general deficits, will be derived using Bayesian modeling. A subset of the participants (who meet the fMRI criteria will be invited to a second session consisting of a multimodal fMRI scan to help determine the functional and structural brain network features of altered gaze perception.

**Sample**

A total of 200 participants, including adolescents (age 14-17) and young adults (18-30), will be recruited. Of the entire sample, approximately 75% will be psychiatric patients (regardless of diagnosis) with various degrees of impaired social functioning and approximately 25% will be demographically matched controls. Demographics will parallel those in the community—approximately 56% White, 29% Black, 6% Asian, and 9% other; of those, approximately 7% Hispanic—and will consist of equal numbers of men and women.

**Methods**

**I. Subject recruitment and assessment**

A. *Subject Recruitment*

Psychiatric patients with varying degrees of social dysfunction and healthy controls will be recruited from several sources. These will include: The OSU Early Psychosis Intervention Center (EPICENTER), word of mouth, researchmatch.org, studysearch, the Simons Foundation Powering Autism Research for Knowledge (SPARK) Project at the Ohio State University, and Internet and community ads. We will also use OSU’s My Chart for Research Recruitment through the Ohio State University’s CTSI. BuildClinical, a research advertising platform, will be utilized to recruit patients with psychosis.

For all participants besides those recruited through BuildClinical an initial screening survey through RedCap will be sent to all interested potential participants. The survey will include a brief description of the study. The survey will be composed of questions designed to help determine eligibility and gather contact and availability information from interested potential participants.

Patients recruited specifically through BuildClinical will be directed to a landing page that contains the option to complete a screening form for themselves or one for their child. They will select the appropriate form and then complete the form. They will not be asked to complete the initial RedCap survey as the BuildClinical screening will be sufficient to determine eligibility and gather contact information.

A telephone screening interview will determine initial eligibility and briefly discuss the risks of the procedure. The voluntary nature of the study will also be stressed, and if the potential participant is interested, an appointment for the assessment interview will be made. No potential subjects will be contacted by projects staff unless they have already provided verbal consent to be contacted (e.g., have called and left a phone message requesting more information about the project).

As part of the consenting process, the study team is seeking permission to video record all assessments administered by the clinical rater for inter-reliability and training purposes. Not all participants will be recorded; this will be determined by needs of the study. Participants may opt out of the recordings during the consent process and remain eligible for the rest of the study. Recordings will be done for ADOS, SANS, MINI, and the Global Functioning: Social and Role assessment.

B. *Inclusion/Exclusion Criteria*

All participants

(1) Age 14 to 30; (2) Ability and willingness to give informed consent; (3) Vision equal to or better than 20/30 on the Snellen visual acuity test, with correction if necessary; (4) No significant neurological abnormalities, such as seizure disorder, mass lesions, etc.; (5) No known Mendelian disorder; (6) No active substance abuse in the 30 days (as determined by the MINI)**; (7) IQ ≥ 80 (based on WASI-II).

*** Subjects who endorse substance use in the last 30 days BUT DO NOT MEET FULL CRITERIA FOR substance use disorder will be given a UDS on the day of the in-person visit and the fMRI visit. Subjects whose UDS indicate the presence of an illicit drug will not undergo that day’s visit. At the PI’s discretion, the participant may be given the option to return at a later date to complete the visit, providing they have a clean UDS at that time. Those that are not given the option will be screen fail.*

Considering significant neurodevelopment in the age range from 14 to 30, we will evenly sample the entire age range (i.e., 1/3 from ages 14-17, 1/3 from ages 18-25, and 1/3 from ages 26-30). This is to ensure a sufficient number of participants across the developing (14-25) and developed (25-30) ranges, enabling statistical analyses to model the *linear* and *quadratic* effects of age in behavioral and brain function.

Patients

(1) A psychiatric condition for which they seek help in a primary care or mental health setting; (2) Significant difficulty in social functioning (sum of the two social items of the WSAS ≥ 4) based on a phone screening administered by the study coordinator or a trained research assistant; and (3) Global Functioning Scale (Social) ≤ 6 or MIRECC Global Assessment of Functioning <70 as assessed by a clinical rater in the interview session.

Additionally, to ensure that the patient sample will contain significant psychopathology traits of interest (psychosis-proneness, autism traits, social anxiety), we will require 60% of the patient sample to meet criteria for a schizophrenia-spectrum disorder, ASD, or social phobia (with at least 20% for each).

Healthy Controls

(1) No history of past or current DSM-5 diagnosis; (2) No social impairment (sum of the two social items of the WSAS < 4) (3) Global Functioning Scale (Social) of ≥7 and ≥70 on MIRECC Global Assessment of Functioning as assessed by a clinical rater in the interview session; and (4) Not taking any psychotropic medications.

fMRI Participants

(1) Ability to tolerate small, enclosed spaces without anxiety; (2) No metals, implants, or metallic substances within or on the body that might cause adverse effects to the subject in a strong magnetic field, or interfere with image acquisition, such as aneurysm clips, retained particles or cerebral stents, CSF stunts, etc.; (3) If a woman of child bearing age, not pregnant or trying to become pregnant; (4) No history of dyslexia.

C. *Assessments*

The assessment protocol comprehensively characterizes participants’ general intellectual functioning, visual perception, social cognition, and social functioning. Eye movements will be recorded using an EyeLink 1000 Plus (SR Research), a video-based eye tracker, during GAZE, JOVI, and TASIT.

Administered by a clinical rater (~120 mins):

1. Mini-International Neuropsychiatric Interview (MINI for DSM-5)** or the MINI Kid and Kid Parent version for participants below age 18 (~30 mins);

*** C-SSRS will be completed in place of the Suicide Assessment Module*

2. Autism Diagnostic Observation Schedule 2^nd^ Edition Module 3 or 4 (ADOS-2) (~40-60 mins);
3. Global Functioning: Social and Role (~20 mins);
4. Scale for the Assessment of Negative Symptoms (SANS) (~10mins);
5. The Mental Illness Research, Education and Clinical Centers Global Assessment Functioning (MIRECC GAF) – Social and Occupational subscales only

Administered by experimenter (~115 mins):

1. Demographic/medical history form (~10 mins)

2) Wechsler Abbreviated Scale of Intelligence Second Edition (WASI-II) (15 mins)

a) Vocabulary

b) Matrix Reasoning

3) Jitter Orientation Visual Integration Task (JOVI) (20 mins)

1. SLOAN Low-Contrast Sensitivity Acuity Test (SLOAN) (10 mins)
2. Eye Gaze Perception Task (GAZE1), Part 1 (~15 mins)
3. The Awareness of Social Inference Test (TASIT) (~20 mins)
4. Snellen Visual Acuity Test (~5 mins)
5. Stanford Acuity Test (StAT) (~5 mins)*
6. Eye Gaze Perception Task (GAZE2), Part 2 (~15 mins) - optional

Completed by participant (~60 mins):

1. Pregnancy screening form (female only)
2. Urine toxicology screen (if required, in case of clinical suspicion of drug abuse; testing done in-person by experimenter using disposable test kit)
3. MRI safety screening form
4. Social Phobia Inventory (SPIN);
5. Work and Social Adjustment Scale (WSAS);
6. Social Anxiety Disorder Dimensional Scale (SAD-D);
7. Autism Spectrum Quotient (AQ);
8. Empathy Quotient (EQ);
9. Cardiff Anomalous Perception Scale (CAPS);
10. Peters Delusion Inventory (PDI);
11. Social Adjustment Scale—Self-Report: Short (SAS-SR: Short)
12. Reading the Mind in the Eyes test version 2 (RME) (10 mins)

Completed by informant* (~10 mins):

1. Social Responsiveness Scale (SRS) adult form or school-age form.

** For children (14-17), informant will be the parent/guardian that signs the consent and accompanies the minor to the visit; For participants 18+, we will ask them to provide us the contact of a family member or a friend who knows them well and can provide information on the participant's social functioning and that individual will serve as the informant. Consent will be obtained from the participant to do this. If consent is not given, they will not be eligible.*

Completed during fMRI scan

1. Whole-brain T1-weighted structural (8 min): 3D SPGR sequence, 0.8mm isotropic resolution, FOV=25.6, TI/TE/FA=1060/Min Full/8°, 320x320 matrix, parallel acceleration factor=2.
2. T2-weighted structural (6 min): Cube sequence, 1mm isotropic resolution, TR/TE= 4100/60 ms, 256x256 matrix; to enable *surface*-based *analysis.*
3. Functional scans: A spin echo-based b0 fieldmap (TR/TE=7400/80ms) with forward and reverse phase encoding will be acquired before the GAZE and again before the resting-state scan, to correct spatial distortion in the images. Functional scans will be acquired using a multi-band slice accelerated (factor=6) gradient echo sequence, 2.4mm isotropic resolution, FOV =23, TR/TE/FA=800/30ms/52˚, 96x96 matrix).

Eye contact perception task (~15 mins): A blocked event-related gaze perception task will be used to probe gaze processing. Face stimuli of the task come from the behavioral version of GAZE described above, but only forward faces will be included in order to keep the task to a reasonable length. Faces are presented in pseudo-randomized order. In the line block, the same face stimuli will be presented with lines over their eyes. Participants indicate whether they feel the face is looking at them or not (yes/no) in the eyes blocks, or if the lines over the eyes are vertical or not (yes/no) in the line blocks. The task will be presented in 3 runs, each run ~5 mins.

Theory of Mind (ToM) task (~15 mins): Participants are presented with a short (2-3 sentence) story, followed by a statement about that story to which they must response ‘true’ or ‘false.’ There are two conditions to the task: BELIEF and PHOTO. In the BELIEF condition, participants are presented with statements about a person and when responding to the subsequent statement, they must take on the perspective of the person in the story to accurately answer the TRUE/FALSE statement. In the PHOTO condition, participants are also presented with brief stories, but this time, they are presented with a statement describing a drawing or photograph and must respond whether the statement is TRUE/FALSE. Both sets of stories require participants to represent false content; the critical difference is in the type of false content represented (i.e., a belief versus a photograph/map). Stories were followed by a true/false question that referred either to the situation in reality or to the false representation.

Resting-state (10 min): Subjects will look at a fixation cross.

4. Diffusion-weighted imaging (11 min): DTI data will be acquired according to the NIMH Adolescent Brain Cognitive Development (ABCD) protocol: multiband factor=3, 102 directions (b=0, 5 volumes; b=500, 6 directions; b=1000, 15 directions; b=2000, 15 directions; b= 3000, 60 directions), 1.7mm resolution, 81 slices, FOV=24, TR/TE=7400mm/minimum, 140x140 matrix.

While scanning parameters may change slightly, power-monitoring software on the scanner will ensure total energy delivered to the subject will remain within FDA guidelines. Specifically, the specific absorption rate (SAR) will be not greater than: (1) 4 W/kg averaged over the whole body for any period of 15 minutes; (2) 3 W/kg averaged over the head for any period of 10 minutes; (3) 8 W/kg in any gram of tissue in the head or torso; (4) 12 W/kg in any gram of tissue in the extremities, for any period of 5 minutes.

**II. Procedure**

Participants will complete up to three study visits. The first visit (lasting between 2 and 3 hours) will take place remotely over a video conference session on a HIPAA-compliant platform. Formal consent will be obtained at the beginning of this visit. This will include a review of an oral assent script [adolescent only], assent from the adolescent, and written consent from their parent/guardian). Electronic informed consent signatures will be collected and (for minors only) a parent/guardian according to BUCK-IRB guidelines through redcap. Then diagnostic interviews, clinical ratings, cognitive assessments, and fMRI screenings if applicable (includes pregnancy screening) will be completed to determine eligibility for the study. Participants deemed eligible will complete self-report surveys and computerized tasks through REDCap. The second visit will last between 2 and 3 hours and take place in 1960 Kenny Roadl. There in-person clinical assessments will be completed by a trained member of staff. Participants will then complete a battery of computerized behavioral tests. For some tasks (Gaze task, TASIT, JOVI), eye tracking data will be collected during task administration.

The third visit is an fmri scanning session that will last about two hours. Contradictions for an MRI scan will be reviewed with the participant orally, and a safety screening form is completed and reviewed to ensure the absence of any contra-indications. Scanning will take place Nation Wide Childrens Hopsital (700 Childrens Drive, Columbus OH 432015) on a 3-Tesla Siemens MRI Scanner that is operated and maintained by the expert staff. Participants’ attention will be monitored through an MR-compatible eye tracker in the scanner. Dr. Lei Wang will provide expert input to optimize scan parameters and supervise fMRI data acquisition. Total time in the scanner, including task and scan setup, is approximately 75 mins.

**III. Statistical Analyses**

The overall analysis plan consists of three main components corresponding to 3 specific aims. Aim 1—we will compare patients/controls on gaze perception metrics (perceptual precision, self-referential bias) and visual scanning patterns during gaze task. Aim 2—we will examine the relationships of gaze perception metrics to clinical and functional measures among patients. Aim 3—we will apply several modeling methods to functional and structural brain data to uncover the neural connectivity architecture underlying altered gaze perception.

*Aim 1. Determine the generality of gaze perception disturbances in psychiatric patients with prominent social dysfunction.*

Two aspects of gaze perception (slope—indexing precision; threshold—indexing self-referential bias) will be derived from the behavioral responses on GAZE and compared between patients and controls using hierarchical Bayesian modeling (HBM).

To determine the effect of visual scanning on gaze perception, eye-tracking data will be analyzed. The number and duration of fixation on the eye region of the face stimuli during GAZE will be compared between the patient and control groups using separate HBMs. The number of fixations will be modeled using a Poisson distribution while duration of fixation modeled using a gamma distribution. We hypothesize that gaze perception disturbances in patients cannot be accounted for by abnormal visual scanning, thus expecting no group differences between number and duration of fixation in the eye region. If group differences in visual scanning exist, then we will re-estimate the group differences in gaze perception metrics by adding visual scanning variables as fixed effect terms to our HBM equations. We expect that after accounting for the individual differences in visual scanning, patients will still show reduced precision and increased self-referential bias compared with controls.

The SLOAN task included in the assessment protocol can serve as a control task, providing a baseline level of contrast sensitivity against which performance can be compared. We will analyze performance on the SLOAN task to estimate each participant’s visual perceptual sensitivity with non-social stimuli. We can then examine if this covaries with social cognition and functioning, and if the slope measure obtained using the gaze perception task is able to explain more variance in psychopathology traits and other social cognitive and functional measures than this non-social slope measure.

*Aim 2: Map behavioral indices of gaze perception disturbances to dimensions of psychiatric phenotypes and core functional domains.*

To obtain more reliable measures of the psychopathology dimensions, we will use principal component analysis (PCA) to extract a common latent variable underlying data generated using different scales or from different sources for each psychopathology dimension. Specifically, a factor score corresponding to psychosis proneness will be extracted from the Peters Delusion Inventory and Cardiff Anomalous Perception Scale; an autism traits factor will be extracted from responses to the ASQ and scores on the ADOS-2; and a social anxiety factor will be extracted from responses to the SPIN and SAD-D. Individual estimate of gaze perception precision obtained in Aim 1 will be correlated with the psychosis proneness and autism traits factors, as well as basic visual perception (SLOAN, JOVI). We hypothesize that gaze perception precision will be negatively associated with psychosis proneness and positively with autism traits, and correlated with basic visual perception functions. Individual estimate of self-referential bias during gaze perception will be correlated positively with social anxiety and psychosis proneness and negatively with autism traits.

Additionally, we will also explore the relationship between the three psychopathology dimensions and gaze perception metrics via model comparison; specifically, we will include the three psychopathology factor scores in the hierarchical Bayesian model in Aim 1 and compare model fit (deviance information criterion, DIC) of the full and reduced models (i.e., with and without the psychopathology dimensions, respectively).

Finally, we will use PCA to extract a common component underlying the three social functioning measures (SAS-SR, S/R functioning, SRS). This social functioning factor will then serve as the dependent variable of a hierarchical regression model, into which the two gaze perception metrics will be entered as predictors one at a time. We expect that gaze perception precision and self-referential bias will account for unique variance in social functioning.

*Aim 3: Identify the neural correlates of altered gaze perception in psychiatric patients with social dysfunction using fMRI.*

**fMRI data pre-processing.** Imaging data (task, rest, and DTI) will be processed in accordance with the Human Connectome Project (HCP) preprocessing pipelines. For task functional data, strict quality control procedures are in place to evaluate movement, excluding runs where the standard deviation of realignment parameters (summed across three rotational axes and three translational directions) is > 0.5mm translation and > 0.2 degrees rotation. At the first-level analysis, 24 motion parameters (6 originals, first derivatives, quadratic of original and derivatives) will be entered as nuisance regressors. For resting-state functional connectivity (rsfcMRI) data, time-series will be band-pass filtered (0.01–0.10 Hz), high motion frames censored, and physiological noise removed using a PCA-based noise correction method (CompCor). DTI data will be manually inspected for significant artifacts as well as undergo an automated quality assessment protocol based on temporal signal-to-noise ratio, mean/maximum voxel intensity outlier count, and mean motion.^78^ Diffusion-weighted scans will be pre-processed and analyzed using FSL 5.0 (FMRIB’s Software Library, www.fmrib.ox.ac.uk/fsl). Scans will be realigned to the b0 image using affine registration and eddy current correction will be applied. DTI analyses must be performed in native space as diffusion gradients are specified in this space; however, regions of interest (ROIs) are created and group analyses will be performed in standard (MNI) space. In order to transform ROIs into each subject’s native space, the T1-weighted structural volume will be realigned to the mean b0-weighted image and subsequently normalized to MNI space. The inverse warping parameters from this step will be used to transform ROIs from MNI space to native space.

**fMRI data analysis.** Analyses of the neuroimaging data will consist of two parts. The first part will examine effective connectivity, functional connectivity, and structural connectivity separately. The second part will use a multivariate method to build predictive models integrating brain connectivity data across modalities.

1) Connectivity:

a) Effective connectivity: Dynamic causal modeling (DCM) will be used to uncover brain dynamics during gaze processing. We will consider a biologically plausible network consisting of 4 volumes of interest (VOIs) informed by our preliminary findings—V2, STS, IPL, and MFC, which are self-connected and connected intrinsically to one another bi-directionally, and V2 will be modeled to receive driving visual input. We will examine how the intrinsic connections and their modulation by gaze discrimination differ between patients and controls. Analyses will be conducted using the HCP CIFTI file format and the associated grayordinates spatial coordinate system. Time-series will be extracted from the 4 VOIs, identified using the Gaze > Gender contrast, and then entered into SPM12 for DCM analyses. Bayesian model averaging (BMA) will be performed to robustly estimate parameters by weighting each possible model’s posterior probability. We hypothesize reduced driving input into V2, weakened feedforward connections from V2, and aberrant gaze modulatory effect on the MFC-V2 feedback connection in patients compared with controls. These 3 parameter estimates will be compared across groups using independent-sample t-tests, corrected for 3 comparisons. These parameters will then be correlated with the 2 gaze perception metrics, to test the hypothesis that reductions in driving input and feedforward connections from V2 will be correlated with reduced gaze perception precision, while aberrant gaze modulation on top-down connections from MFC will be correlated with more self-biased gaze perception.

b) Resting-state functional connectivity: Cortical surface and subcortical regions, in grayordinates space, will be parcellated based on the Gordon et al. and Seitzman et al. parcellation schemes, and assigned to 14 networks highly resembling the Power networks.^84^ Pearson’s product-moment correlation coefficients will then be calculated pairwise between time courses for each of the parcels, producing connectivity matrices for all subjects. Fisher’s r-z transformation will be applied. Group comparisons will focus on intra-network connectivity within the Visual Network (VisN) and inter-network connectivity between the Vis, cingulo-opercular, dorsal attention, and salience networks. We expect patients to show reduced intra-VisN connectivity, associated with reduced precision in gaze perception, and aberrant inter-network connectivity, associated with self-referential bias.

c) Structural connectivity: We will use probabilistic tractography to assess anatomical pathways between V2, STS, IPL, and MFC—identified using the method described in the DCM analyses above and converted from surface space (CIFTI) to volume space (NIFTI). Probabilistic tractography will be run between the ROIs within the visual system (V2, STS, IPL) and between these visual ROIs and the frontal ROI (MFC), resulting in 6 total tracts, following methods used by Co-I Thakkar in previous studies. Single-subject results for each of the probabilistic tracts will be transformed to MNI space in order to perform group analyses. For each subject, mean fractional anisotropy, mean diffusivity, axial diffusivity, and radial diffusivity within each of the group-thresholded tracts will be extracted. These measures are thought to reflect different aspects of the biological microstructure. We will compare these measures between the patient and control groups, and examine their correlations with the 2 gaze perception metrics and 3 psychopathology dimensions.

2) Multimodal Predictive Model:

a) We will apply the Brain Based Set (BBS) method to identify components integrating brain information across modalities that best predict gaze perception performance and social functioning. Effective connectivity, rsfc, and DTI measures will be input as predicting variables to identify components that best predict the gaze perception metrics (slope, threshold) and social functioning. A 10-fold cross-validation will be used.

If we fail to detect significant relationships between clinical features and behavioral/brain measures, we will use an alternative approach, sparse canonical correlation analysis, which uses a machine-learning approach to discover a linear combination of the clinical features that maximally correlates with a linear combination of the behavioral and brain measures. This approach penalizes the number of dimensions of the data to minimize over-fitting. It has been applied successfully in multiple neuroimaging studies.

**IV. Payment**

Participants can earn up to $180:

Visit 1a – Remote Screening Visit AND

Visit 1b – In-Person Testing Visit	$ 95.00 **

Additional computer task (optional)	10.00

Visit 2 - fMRI Visit	75.00

**TOTAL**	**$ 180.00**

***Those found to be ineligible to participate after the interview portion of the assessment visit will still be compensated a total of $20 for their time.*

Payments will be paid via Greenphire ClinCard which is a reloadable debit card. SRS informants will also receive a one-time payment of a $10 gift card for completing their questionnaires.

**V. Protection of Human subjects**

A. *Risk assessment*

Known or expected risk for all participants:

(1) There is a risk of discomfort or boredom when being asked questions about mental health symptoms, substance use, or completion of tasks.

Known or expected risk for participants of fMRI study:

(1) Discomfort or anxiety from being in the confined space of the MRI scanner
(2) Fast imaging sequences, such as those employed in this study, have the potential to induce peripheral nerve stimulation (PNS). PNS can be described as a light touching sensation on the skin surface and may cause mild discomfort, but is not harmful to the subject
(3) Risks of hearing damage due to loud noises produced by the scanner
(4) Risk that the MRI scan will reveal a minor or significant lesion in the brain (e.g.,a tumor) previously unknown to the subject and requiring additional follow-up
(5) Risk of injury from objects accelerated by the strong magnetic field of the magnet, striking the subjects, or metallic substances on the skin or foreign bodies implanted deliberately or accidentally in the subject that acquire kinetic or thermal energy from the magnetic or radiofrequency emissions of the MRI, causing tissue injury to the subject
(6) Sometimes, subjects report a temporary, slight dizziness or light-headedness when they come out of the scanner
(7) Potential risk for pregnant women.

B. *Minimization of risk*

Individuals will be instructed that they do not have to answer questions that make them uncomfortable. They can also choose to discontinue their participation for any reason at any time. Clinically trained study staff will be alert for the emergence of new suicidal thoughts amongst enrolled subjects during the course of participation. In the event of concerns about suicide risk (revealed during the assessment phase), the PI will be promptly notified, and plans will be formulated for additional emergency evaluation at the psychiatric emergency room, if appropriate. Participants can also elect to not participate in or discontinue the MRI scan by pressing a button in the machine at any time. Participants who elect to participate in the MRI scan will complete an MRI safety screening to make sure they don’t have medical conditions or foreign objects in the body that are contraindicative of MRI. Discovery and disclosure of incidental finding or abnormality on MRI scans: During the formal consent process, all subjects will be informed about the potential risks of discovering an incidental finding or abnormality on their MRI scan. MRI scans acquired in this protocol will be checked for incidental findings according to the procedure established by Nationwide Children’s Hospital. Any incidental findings deemed significant will be brought to the attention of the participant and consultation with their primary care provider will be available.

C. *Disposition of drop outs and withdraws*

When participants drop out during the assessment, but before enrollment, all data including consent form will be destroyed. However, if a participant would agree to be re-contacted for future studies, the consent form will be retained. Limited, de-identified data regarding ethnicity, age, gender, and diagnosis will be retained through the screening process in order to establish the sampling frame. If a participant drops out due to substance use but would otherwise meet the inclusion criteria, all data will be retained until the closing of the project, in case the participant becomes eligible and wishes to be reconsidered for the study before conclusion of recruitment. When participants drop out after enrollment, data may be destroyed, depending upon the wishes of the participant. However, consent forms and the research number will be retained.

D. *Data Safety and Monitoring Plan*

All demographics and clinical data will be stored online in the HIPAA-compliant REDCAP data capture system. Participant's names will not be entered into the REDCAP system. Rather, a subject number will be used to identify subjects on assessment materials. Each subject is given a unique number and there is a password-protected master key database separate from study data linking the subject identity with the code, stored in secure password-protected OSU server only accessible to OSU research team members. The masterkey file linking subjects’ identities to the research numbers will be destroyed 6 years after completion of the study, thereby anonymizing the data. Survey data will be collected from participants using OSU’s REDCap platform; each participant will be sent a unique link to their survey and no identifiable information will be collected on REDCap. Paper forms with the participant code will be kept in a locked cabinet in the Department of Psychiatry where only official study staff has access. Data collection from computerized and fMRI tasks for each participant will be saved with a research identifier number only and stored in

computer files without reference to any personally identifiable information. These files will be stored on a local OSU server that is username and password protected. All research materials will be maintained in strict confidentiality. Only official OSU study staff have access to the data. If any member of the research study leaves, his or her access to the data will be removed immediately.

Adverse event reporting will occur on the standard BUCK-IRBtimetable for the Baseline Assessments and MRI scanning session.

Data sharing

The value of this research is significantly enhanced by making the data available for the widest possible use, and because of the rapidly changing world of data analysis, it is impossible to predict what future research will do with data collected for any particular project in the present. By keeping the data indefinitely, it will become available for projects, yet to be determined. These include projects run by the investigators, as well as those outside the institution through data sharing plans or central repositories. The NIMH, which funds this study, requires that data be uploaded into the NIMH Data Archive (NDA), which is a requirement of receiving funding.

When data are transferred to co-investigators and data repositories, de-identification will be performed, removing all HIPAA identifiers from the files. To prevent reconstruction of facial likenesses from structural image datasets, we will blur/obscure/strip the information from the MRI image around the face.

Subjects will be informed in the consent document that the data will be stored and that it may be shared with other investigators, both at the University and outside the University, through central databanks. The risk posed to subjects will be very, very small. Storing the data for an indefinite amount of time will follow strict protocols, outlined above, that will ensure the safety of the data and minimize the risk of accidental disclosure. Data that is shared or uploaded to central databanks will be de-identified and will be effectively anonymized, since investigators using these central databases will not have access to the keys linking individual subjects to the data. Furthermore, no data will be shared without an agreement by stewards of the central databank to require data use agreements and prevent investigators from even attempting to identify subjects. It is extremely unlikely that an investigator would even be able identify subjects from shared data, without already having access to a subject's identifiers.
