# Supplementary material for: Sex differences in social cognition: Insights from a sample enriched for social dysfunction
Source: PLoS One. 2026 Sep 10;21(9):e0354796. doi: 10.1371/journal.pone.0354796 (PMC13561320; doi:10.1371/journal.pone.0354796)
Supplement: Supplementary file 4 [file pone.0354796.s004.docx]

**S4 Text. Description of psychopathology measures.**

The Peters Delusion Inventory (PDI; Peters et al., 1998), Cardiff Anomalous Perception Scale (CAPS; Bell et al., 2006), and Scale for the Assessment of Negative Symptoms (SANS; Andreasen, 1984) were administered to capture psychosis proneness. The PDI is a 21-item, self-report measure that captures the extent to which an individual experiences delusional ideation (e.g., referential beliefs). The CAPS is a 32-item, self-report measure that captures the extent to which an individual experiences perceptual aberrations, such as illusions or hallucinations. For both the PDI and CAPS, the total number of items endorsed “yes” were summed to provide a total score on each scale. The SANS is an interviewer-rated measure of negative symptoms that are characteristic of psychosis-spectrum disorders. Trained interviewers rated participants on four negative symptoms (i.e., affective flattening, alogia, avolition-apathy, anhedonia-asociality) on a scale of 0=absent to 5=severe. Items were summed to provide an overall rating of negative symptom severity.

To examine autism spectrum traits, the Autism Spectrum Quotient (AQ; Baron-Cohen et al., 2001b) and the Autism Diagnostic Observation Schedule (ADOS; Hus & Lord, 2014) were administered. The AQ is a 50-item self-report measure in which participants rate their level of agreement with each item on a scale of “definitely disagree” to “definitely agree”. Responses reflecting slight or definite endorsement of an autism spectrum trait were coded as “1”, and ratings were summed to provide a total score. The ADOS is semi-structured, interviewer-rated measure in which a trained interviewer assesses participants’ behaviors in the context of socially engaging tasks. The interviewer rates items characterizing participants’ communication, reciprocal social interaction, imagination, and stereotyped behaviors and restricted interests, which are then summed using the revised algorithm (Hus & Lord, 2014). For both the AQ and ADOS, higher values indicate greater levels of autism spectrum traits.

To examine social anxiety symptoms, the Social Phobia Inventory (SPIN; Connor et al., 2000) and Social Anxiety Disorder Dimensional Scale (SAD-D; LeBeau et al., 2012) were administered. The SPIN is a 17-item self-report measure in which participants rate items on a scale of 0=not at all to 4=extremely. The SAD-D is a 10-item self-report measure in which participants rate the extent to which they have experienced DSM-5 social anxiety disorder symptoms in the past month on a scale of 0=never to 4=all of the time. For both the SPIN and SAD-D, higher scores indicate greater social anxiety.

Inclusion of multiple measures increases reliable measurement of these dimensions, as each measure targets nuanced features. For instance, the SANS, PDI, and CAPS all capture distinct symptom categories characteristic of psychosis proneness, with the SANS capturing the “deficit” experiences, PDI capturing delusional ideation, and CAPS capturing hallucinations or perceptual aberrations. The ADOS and AQ both assess autism spectrum traits; however, the ADOS is a standardized interview assessment, whereas the AQ is a self-report measure. Therefore, these measures capture both externally and internally experienced signs of autism which are necessary for a comprehensive characterization of this dimension. Lastly, the SAD-D and SPIN are both self-report measures of social anxiety traits, yet they capture slightly different phenomenology. The SAD-D captures symptoms that specifically map onto DSM-5 criteria of social anxiety disorder, whereas the SPIN captures a broader range of social anxiety experiences that are more evenly distributed across the general population. Thus, inclusion of both self-report measures appears to provide broad coverage of this trait dimension than that offered by either measure alone.
