# Supplementary material for: Sex differences in social cognition: Insights from a sample enriched for social dysfunction
Source: PLoS One. 2026 Sep 10;21(9):e0354796. doi: 10.1371/journal.pone.0354796 (PMC13561320; doi:10.1371/journal.pone.0354796)
Supplement: Supplementary file 5 [file pone.0354796.s005.docx]

**S5 Text. Gaze Task psychometric curve fitting.**

To accommodate differences in perceptual sensitivity for processing gaze of forward and deviated faces, the gaze angle ranges for the two head orientation conditions were adjusted. Specifically, forward faces had gaze angles from 0° to 10° in increments of 1.25°, whereas deviated faces had gaze angles from 0° to 24° in increments of 3°. We used a larger range of gaze angles for deviated faces because, in pilot testing, using the 0°–10° range resulted in >50% endorsement even for the most averted gaze (10°), which dramatically shifted the psychometric curve. Using 0°–24° for deviated faces yielded more centered data and therefore more valid estimations of gaze perception metrics.

We used the psignifit 4 toolbox (Schütt et al., 2016) implemented in MATLAB for Bayesian estimation of perceptual precision during gaze perception. To enable comparable mathematical operations between head orientation conditions, gaze angles were first converted to a scale of “eye-contact signal strength” ranging from 0 (completely averted) to 1 (completely direct) in increments of 0.125. For each participant, a logistic psychometric curve was fit to endorsement rates at each eye-contact signal strength, separately for forward and deviated faces.

The psignifit 4 toolbox uses a 4-parameter psychometric function that estimates threshold (*m*), width (*w*), lapse rate (λ), and guess rate (γ). Because the task design forces participants to provide a response on each trial, lapse and guess rates are generally near zero. However, to account for rare instances of chance performance and button press errors, we fixed the lapse rate and guess rate at 2% to improve curve fit (Kingdom & Prins, 2010). As a result, the 4-parameter equation essentially becomes a 2-parameter equation estimating only the parameters for threshold and width, with γ and λ being constants:

ψ(x | m, w, γ, λ) = γ + (1 − γ − λ) · 1 / (1 + e^−2ln(1/0.05 − 1) · (x − m)/w^)

where γ = 0.02 and λ = 0.02.

For the present study, we focused on the width parameter (*w*) as our index of gaze perception. Width captures perceptual precision, measuring sensitivity to small deviations in eye gaze direction across trials. As such, width primarily reflects low-level, sensory-based encoding of gaze information and is less influenced by decisional or interpretive processes. Our study aims to isolate an intermediate perceptual stage linking basic visual processing with downstream social cognitive outcomes; therefore, width is the most appropriate measure.

In contrast, threshold reflects the point at which gaze is judged as self-directed and has been shown to index self-referential bias—the tendency to endorse eye contact. This judgment relies on higher-order cognitive processes, including prior knowledge, expectations, and beliefs about social intent, rather than purely sensory encoding of gaze direction.
