# Supplementary material for: Sex differences in social cognition: Insights from a sample enriched for social dysfunction
Source: PLoS One. 2026 Sep 10;21(9):e0354796. doi: 10.1371/journal.pone.0354796 (PMC13561320; doi:10.1371/journal.pone.0354796)
Supplement: Supplementary file 6 [file pone.0354796.s006.docx]

| Criterion Variable | No Demographic Covariates | | | |  | | | Covarying Age, IQ | | | |
| --- | --- | --- | --- | --- | --- | --- | --- | --- | --- | --- | --- |
|  | *b* | *β* | *t* | *p* | |  | *b* | | *β* | *t* | *p* |
| **Gaze-Eyes** | **.19** | **.25** | **2.63** | **.010*** | |  | **.20** | | **.24** | **2.80** | **.006**** |

*Note.* Sex was used as independent variable (females = 0, males = 1). This model did not include the Lines condition as a covariate.
