# Supplementary material for: Sex differences in social cognition: Insights from a sample enriched for social dysfunction
Source: PLoS One. 2026 Sep 10;21(9):e0354796. doi: 10.1371/journal.pone.0354796 (PMC13561320; doi:10.1371/journal.pone.0354796)
Supplement: Supplementary file 7 [file pone.0354796.s007.docx]

|  | Social Affect | Psychosis-Proneness | Low Social Reciprocity |
| --- | --- | --- | --- |
| Social Phobia Inventory | .90 | .20 | .09 |
| Social Anxiety Disorder Dimensional Scale | .84 | .29 | .15 |
| Autism Spectrum Quotient | .78 | .03 | .35 |
| Autism Diagnostic Observation Schedule | .18 | -.10 | .90 |
| Scale for the Assessment of Negative Symptoms | .21 | .33 | .79 |
| Cardiff Anomalous Perception Scale | .24 | .89 | .05 |
| Peters Delusion Inventory | .13 | .93 | .09 |
| Total Variance | 33% | 27% | 23% |
