# Supplementary material for: Sex differences in social cognition: Insights from a sample enriched for social dysfunction
Source: PLoS One. 2026 Sep 10;21(9):e0354796. doi: 10.1371/journal.pone.0354796 (PMC13561320; doi:10.1371/journal.pone.0354796)
Supplement: Supplementary file 8 [file pone.0354796.s008.docx]

|  | **Female** | | **Male** | |  |  |
| --- | --- | --- | --- | --- | --- | --- |
|  | *M* | *SD* | *M* | *SD* | *t* | *p* |
| Social Affect | .16 | 1.04 | -.25 | .87 | **2.88** | **.004**** |
| Psychosis Proneness | -.08 | .91 | .13 | 1.12 | -1.30 | .196 |
| Low Social Reciprocity | -.22 | .93 | .35 | 1.02 | **-3.80** | **<.001***** |

* *p* < .05

** *p* < .01
