# Supplementary material for: Sex differences in social cognition: Insights from a sample enriched for social dysfunction
Source: PLoS One. 2026 Sep 10;21(9):e0354796. doi: 10.1371/journal.pone.0354796 (PMC13561320; doi:10.1371/journal.pone.0354796)
Supplement: Supplementary file 9 [file pone.0354796.s009.docx]

| Criterion Variable |  | Covarying Age, IQ, Psychopathology Factors | | | |
| --- | --- | --- | --- | --- | --- |
|  | *n* | *b* | *β* | *t* | *p* |
| Gaze Perception Task – No Scanner |  |  |  |  |  |
| Gaze-Forward | 166 | .03 | .03 | .40 | .687 |
| **Gaze-Deviated** | **166** | **.11** | **.20** | **2.49** | **.014*** |
| Gaze Perception Task – Scanner |  |  |  |  |  |
| **Gaze-Eyes** | **102** | **.19** | **.34** | **2.74** | **.007**** |
| Gaze-Lines | 102 | .01 | -.13 | .12 | .909 |
| **Reading the Mind in the Eyes** | **181** | **-1.54** | **-.19** | **-2.38** | **.019*** |
| The Awareness of Social Inference Test | 163 | -.02 | -.09 | -1.09 | .278 |

* *p* < .05

** *p* < .01

*Note.* Sex was used as independent variable (females = 0, males = 1). 1. When predicting performance on Gaze-Eyes, we controlled for performance on Gaze-Lines (but not vice versa). All three psychopathology factors were entered simultaneously as covariates. **Lines in bold represent a significant sex difference (favoring females).**
